# Association between dental health and obstructive coronary artery disease: an observational study

**DOI:** 10.1186/s12872-019-1080-9

**Published:** 2019-04-27

**Authors:** Ho Lee, Hack-Lyoung Kim, Kwang Nam Jin, Sohee Oh, Yoon-Sic Han, Da-Un Jung, Hye-Young Sim, Hee-Sun Kim, Woo-Hyun Lim, Jae-Bin Seo, Sang-Hyun Kim, Joo-Hee Zo, Myung-A Kim

**Affiliations:** 1 Section of Dentistry, Seoul Metropolitan Government-Seoul National University Boramae Medical Center, Seoul, South Korea; 2 Division of Cardiology, Department of Internal Medicine, Seoul Metropolitan Government-Seoul National University Boramae Medical Center, Seoul National University College of Medicine, 20, Boramae-ro 5-gil, Dongjak-gu, Seoul, 07061 South Korea; 3 Department of Radiology, Seoul Metropolitan Government-Seoul National University Boramae Medical Center, Seoul National University College of Medicine, Seoul, South Korea; 4 Department of Biostatistics, Seoul Metropolitan Government-Seoul National University Boramae Medical Center, Seoul National University College of Medicine, Seoul, South Korea

**Keywords:** Coronary artery disease, Dental health, Inflammation, Tooth loss

## Abstract

**Background:**

The association between dental health and coronary artery disease (CAD) remains a topic of debate. This study aimed to investigate the association between dental health and obstructive CAD using multiple dental indices.

**Methods:**

Eighty-eight patients (mean age: 65 years, 86% male) were prospectively enrolled before undergoing coronary CT angiography (*n* = 52) or invasive coronary angiography (*n* = 36). Obstructive CAD was defined as luminal stenosis of ≥50% for the left main coronary artery or ≥ 70% for the other epicardial coronary arteries. All patients underwent thorough dental examinations to evaluate 7 dental health indices, including the sum of decayed and filled teeth, the ratio of no restoration, the community periodontal index of treatment needs, clinical attachment loss, the total dental index, the panoramic topography index, and number of lost teeth.

**Results:**

Forty patients (45.4%) had obstructive CAD. Among the 7 dental health indices, only the number of lost teeth was significantly associated with obstructive CAD, with patients who had obstructive CAD having significantly more lost teeth than patients without obstructive CAD (13.08 ± 10.4 vs. 5.44 ± 5.74, *p* < 0.001). The number of lost teeth was correlated with the number of obstructed coronary arteries (*p* < 0.001). Multiple binary logistic regression analysis revealed that having ≥10 lost teeth was independently associated with the presence of obstructive CAD (odds ratio: 8.02, 95% confidence interval: 1.80–35.64; *p* = 0.006).

**Conclusions:**

Tooth loss was associated with the presence of obstructive CAD in patients undergoing coronary evaluation. Larger longitudinal studies are needed to determine whether there is a causal relationship between tooth loss and CAD.

## Background

Coronary artery disease (CAD) is a leading cause of death worldwide, and is associated with a major socioeconomic burden [1].Well-known factors that are associated with CAD include hypertension, diabetes mellitus, dyslipidaemia, and smoking [2], however; these traditional risk factors cannot fully explain the clinical features and increasing burden of CAD [3]. Thus, researchers are seeking to identify additional risk factors for atherosclerotic cardiovascular disease. Recent results have indicated that chronic inflammation may contribute to the development and progression of atherosclerosis [4, 5], with dental infection being a possible cause of chronic inflammation, as the oral cavity is a major site of chronic infection and inflammation, especially in cases of periodontal disease. Many studies have revealed that the long-standing inflammatory stimuli of dental infection is involved in the pathogenesis of atherosclerosis and subsequent CAD [5–14], although other studies have failed to detect a clear association between dental infection and CAD [15–17]. Thus, there remains debate regarding the association between dental infection and CAD.

The high prevalence rates of both periodontal disease and CAD have highlighted the importance of quantifying their association to improve public health. Although there are several types of dental indices that reflect different aspects of dental health, most previous studies have only focused on 1 or 2 dental indices. Therefore, the present study aimed to investigate the association between dental health and CAD using multiple dental indices.

## Methods

### Participants

This single-centre observational study was performed at the Seoul Metropolitan Government-Seoul National University Boramae Medical Center (Seoul, Korea). Between February 2013 and January 2015, patients with suspected CAD were recruited before undergoing coronary computed tomography angiography (CCTA) or invasive coronary angiography (CAG). Patients were excluded if they had: 1) non-dental chronic inflammatory conditions, 2) recently used antibiotics or anti-inflammatory drugs, 3) acute myocardial infarction or coronary revascularisation within 6 months, 4) acute decompensated heart failure, or 5) history of osteomyelitis of jaw, facial trauma accompanied by tooth injury, or maxillofacial tumour. The study’s protocol was approved by the institutional review board of Boramae Medical Center (# 16–2013-34) and complied with the tenets of the 1964 Declaration of Helsinki and its later amendments. Informed consent was obtained from all participants.

### Clinical data collection

We obtained information regarding the participants’ demographic characteristics (age and body mass index [BMI, kg/m^2^]) and traditional risk factors (hypertension, diabetes mellitus, dyslipidaemia, ischaemic heart disease, and smoking status). Hypertension was identified based on (1) a history of hypertension, (2) anti-hypertensive medication, or (3) a resting systolic blood pressure of ≥140 mmHg or a resting diastolic blood pressure of ≥90 mmHg. Diabetes mellitus was identified based on (1) a history of diabetes, (2) anti-diabetic medication, or (3) a fasting glucose level of ≥126 mg/dL. Dyslipidaemia was identified based on (1) a history of dyslipidaemia, (2) anti-dyslipidaemic medication, or (3) a total cholesterol level of ≥200 mg/dL or a low-density lipoprotein (LDL) cholesterol level of ≥130 mg/dL. Current smoking was defined as regularly smoking cigarettes during the last 12 months. The pre-test probability of CAD was determined based on age, sex, and the nature of chest pain during the initial presentation, with the results classified as low (< 10%), intermediate (10–90%), or high (> 90%) [18].Venous blood samples were obtained for laboratory testing after an 8-h overnight fast. The laboratory tests included white blood cell count, haemoglobin, total cholesterol, LDL and high-density lipoprotein (HDL) cholesterol, triglycerides, fasting glucose, glycated haemoglobin, and serum creatinine. The estimated glomerular filtration rate was calculated as 175 × serum creatinine^− 1.154^ × age^− 0.203^(and × 0.742 if the patient was a woman).

### Assessment of CAD

The presence of CAD was assessed using CCTA (*n* = 52) or invasive CAG (*n* = 36). The CCTA protocol has been previously reported [19, 20]. It is based on CT scans for scoring coronary artery calcium and retrospectively ECG-gated coronary CT angiography. The scans were performed using a 128-slice CT scanner (Ingenuity; Philips Medical Systems, the Netherlands). Patients with a heart rate of ≥65 beats/min received bisoprolol (5–10 mg orally) 1 h before the examination if there was no contraindication. Sublingual nitro-glycerine (0.6 mg) was routinely used immediately before starting the CT scan. Image acquisition was performed in the cranio-caudal direction. To estimate the degree of stenosis, we visually traced the coronary lumen at the maximal stenotic portion and compared it to the mean value from proximal and distal reference segments. Cardiac catheterisation was performed using the standard technique [21], and used to assess the degree of epicardial coronary artery stenosis. Obstructive CAD was considered present based on luminal stenosis of ≥ 50% for the left main coronary artery or ≥ 70% for the other epicardial coronary arteries, and the extent of CAD was classified as 1-, 2-, or 3-vessel disease. After the CCTA and invasive CAG, all management strategies for the CAD, including coronary revascularisation and medication, were selected at the discretion of attending physician.

### Quantification of dental health status

The dental health indices included the dental caries indices, the periodontitis indices, and comprehensive dental indices. The dental caries indices were the sum of decayed and filled teeth (DFT) and the ratio of no restoration (RNR) [22]. The DFT was defined as the total number of teeth that had caries or had been previously treated for caries. The RNR was calculated as the ratio of the number of decayed teeth to the DFT. The periodontitis indices were the community periodontal index of treatment needs (CPITN) and clinical attachment loss (CAL) [22]. The periodontal pocket depth was measured using the World Health Organisation periodontal probe (Qulix™ Color-Coded Probes – PCP-11.5B WHO, Single end; Hu-Friedy Mfg Co. Inc., Chicago, IL, USA). The dental arch was classified into six areas: upper/lower posterior teeth, right/left posterior teeth, and upper/lower anterior teeth. In each area, the tooth with the most severe periodontal disease was assigned a score of 0–4 (0: healthy periodontal status without bleeding on probing, calculus, or a periodontal pocket; 1: bleeding on probing without calculus or a periodontal pocket; 2: presence of calculus with a periodontal pocket depth of < 3.5 mm; 3: presence of a periodontal pocket depth of 3.5–5.5 mm; 4: presence of a periodontal pocket depth of > 5.5 mm depth). The largest value among these areas was defined as the CPITN, although if < 2 teeth were present in an area, that area was combined with the adjacent area. The presence of CAL was defined based on the distance from the cementoenamel junction to the junctional epithelium attachment of each tooth. The tooth with the highest CAL in each area was scored and the final CAL was defined as the average of all 6 areas (0: < 3.5 mm, 1: 3.5–5.4 mm, 2: 5.5–8.4 mm, 3: 8.5–11.4 mm, 4: ≥11.5 mm). The comprehensive dental indices included the total dental index (TDI), the panoramic topography index (PTI), and the number of lost teeth [22]. The TDI was calculated the sum of scores related to dental caries (0: no caries, 1: 1–3 caries lesions, 2: 4–7 caries lesions or unimaxillary edentulism, 3: > 8 caries lesions, residual roots, or bimaxillary edentulism), periodontitis (0: none, 1: periodontal pocket depth of 4–5 mm, 2: periodontal pocket depth of > 6 mm, 3: macroscopic pus in the periodontal pocket), periapical lesions (0: none, 1: 1 lesion or vertical bone pocket, 2: 2 lesions, 3: ≥3 lesions), and pericoronitis (0: absent, 1: present) [8, 12, 13]. The PTI was calculated as the sum of the number of teeth, excluding wisdom teeth, with various pathological states: periapical lesion or cyst, vertical periodontal pocket depth of > 4 mm, furcation involvement, unrestorable dental caries, residual root, pericoronitis with a follicular space width of > 3 mm [7, 23].

### Statistical analysis

Continuous variables were expressed as mean ± standard deviation and categorical variables were expressed as number (percentage). The clinical characteristics of patients with and without CAD were compared using the chi-square test for categorical variables or Student’s *t* test for continuous variables. Inter-group comparisons of mean values for a variable according to CAD extent were performed using analysis of variance (ANOVA). The optimal cut-off values for predicting obstructive CAD were identified using receiver operating characteristic (ROC) curve analysis. Multivariable logistic regression analysis was performed to identify factors that predicted obstructive CAD independent of age, sex, BMI, diabetes mellitus, and smoking status. The Hosmer-Lemeshow test was performed to test for goodness of fit for the logistic regression model. Differences were considered statistically significant at a *p-*value of < 0.05, and all analyses were performed using IBM SPSS software (version 22.0; IBM Corp., Armonk, NY, USA).

## Results

The 88 included patients were mainly men (86.4%) and had a mean age of 64.8 ± 7.7 years. Forty patients (45.4%) had obstructive CAD and the patients’ characteristics according to obstructive CAD status are shown in Table 1. Both groups had similar mean ages, although patients with CAD were more likely to be men, to have a lower BMI, to have diabetes, and to be current smokers. Most patients in both groups had intermediate pre-test probabilities, and patients with obstructive CAD had higher laboratory results for fasting glucose.

**Table 1:** Clinical characteristics of study patients

Characteristic	With CAD (*n* = 40)	Without CAD (*n* = 48)	*p*
Age, years	65.3 ± 7.6	63.3 ± 7.4	0.247
Male sex, n (%)	38 (95.0)	38 (79.2)	0.031
Body mass index, kg/m^2^	23.6 ± 3.5	25.2 ± 3.4	0.047
Risk factors, n (%)			
Hypertension	21 (52.5)	28 (53.8)	0.583
Diabetes mellitus	14 (35.0)	5 (10.4)	0.005
Dyslipidaemia	12 (30.0)	10 (20.8)	0.323
Current smoking	13 (33.3)	2 (4.3)	< 0.001
Pretest probability, n (%)			0.110
Low	0	3 (6.2)	
Intermediate	35 (87.5)	43 (89.6)	
High	5 (12.5)	2 (4.2)	
Laboratory findings			
White blood cell count, /*μ*L	7351 ± 2661	6825 ± 2058	0.302
Haemoglobin, g/dL	13.7 ± 1.6	13.4 ± 2.1	0.495
Total cholesterol, mg/dL	169 ± 0	173 ± 40	0.687
LDL cholesterol, mg/dL	99 ± 42	110 ± 38	0.246
HDL cholesterol, mg/dL	43.4 ± 11.8	48.7 ± 13.0	0.077
Triglyceride, mg/dL	130 ± 100	133 ± 72	0.901
Fasting glucose, mg/dL	122 ± 44	104 ± 17	0.016
Glycated haemoglobin, %	6.63 ± 1.64	5.97 ± 0.69	0.053
Estimated GFR, mL/min/1.73m^2^	82.6 ± 19.8	85.5 ± 16.4	0.466

*CAD* coronary artery disease, *LDL* low-density lipoprotein, *HDL* high-density lipoprotein, *GFR* glomerular filtration rate

**Fig. 1:**
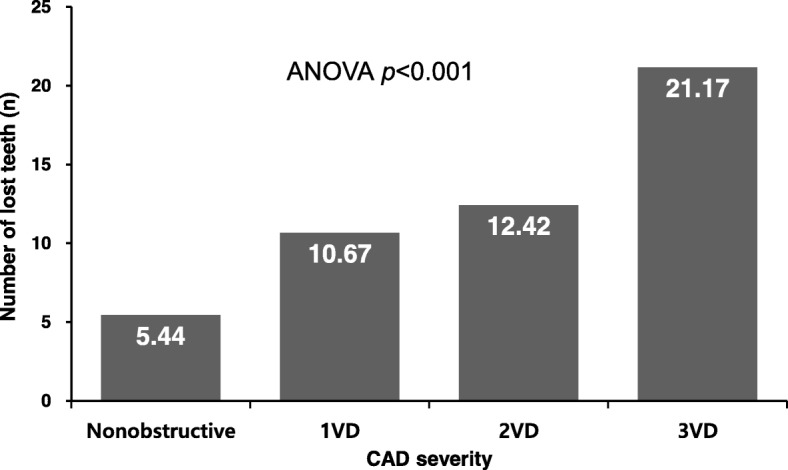
Linear association between CAD severity and number of lost teeth ANOVA, analysis of variance; VD, vessel disease; CAD, coronary artery disease

**Fig. 2:**
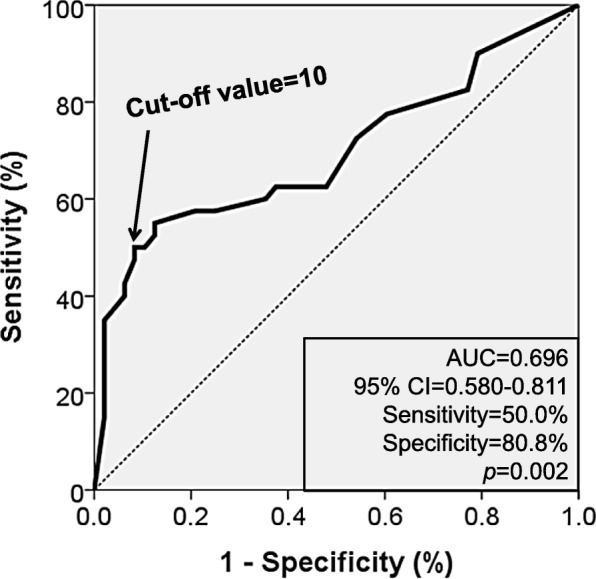
ROC curve analysis showing the cut-off value for number of lost teeth to predict obstructive coronary artery disease ROC, receiver-operating characteristic; AUC, area under curve; CI, confidence interval.

The results of the dental examinations are shown in Table 2. The only significant difference between the groups with and without obstructive CAD was observed for tooth loss, with obstructive CAD being associated with a significantly higher mean number of lost teeth (13.08 ± 10.4 vs. 5.44 ± 5.74, *p* < 0.001). Furthermore, increasing CAD severity was significantly correlated with a larger number of lost teeth (ANOVA *p* < 0.001) (Fig. 1). The ROC curve analysis identified 10 lost teeth as the optimal cut-off value (area under curve: 0.696, sensitivity: 50.0%, specificity: 80.8%) (Fig. 2). The multiple binary logistic regression analysis revealed that having ≥10 lost teeth independently predicted the presence of obstructive CAD (odds ratio: 8.02, 95% confidence interval: 1.80–35.64; *p* = 0.006) (Hosmer-Lemeshow test, *p* = 0.825) (Table 3).

**Table 2:** Results of dental health examinations

Characteristic	With CAD (*n* = 40)	Without CAD (*n* = 48)	*p*
*Dental caries index*			
Sum of decayed teeth and filled teeth (DFT)	5.22 ± 4.09	6.88 ± 4.10	0.065
Ratio of no restoration (RNR)	0.20 ± 0.33	0.27 ± 0.32	0.273
*Periodontitis index*			
Community periodontal index of treatment need (CPITN)	3.16 ± 1.29	3.42 ± 1.03	0.307
Clinical attachment loss (CAL)	1.37 ± 0.96	1.14 ± 0.63	0.179
*Comprehensive index*			
Total dental index (TDI)	3.75 ± 1.62	3.75 ± 1.72	0.999
Panoramic topography index (PTI)	12.25 ± 7.98	13.30 ± 7.52	0.529
Number of lost teeth	13.08 ± 10.4	5.44 ± 5.74	< 0.001

*CAD* coronary artery disease

**Table 3:** Independent predictors of obstructive coronary artery disease

Variable	Odds ratio	95% confidence interval	*p*
Age ≥ 65 years	0.86	0.24–3.07	0.818
Body mass index≥25 kg/m^2^	1.60	0.44–5.84	0.474
Diabetes mellitus	1.82	0.40–8.25	0.432
Smoking	5.23	2.03–13.45	0.001
Lost teeth≥10	8.02	1.80–35.64	0.006

## Discussion

The present study revealed that, among various dental health indices, only the number of lost teeth (which reflects the most severe outcome of sustained oral infection) was independently associated with obstructive CAD. Furthermore, the extent of CAD increased proportionally with an increasing number of lost teeth. However, obstructive CAD was not clearly associated with any of the other dental indices, which included the DFT, RNR, CPITN, CAL, TDI, and PTI.

### Association between dental health and CAD

Many studies have indicated that dental health is associated with the development of atherosclerotic cardiovascular disease [5–14]. For example, a case-control study by Mattila et al. revealed that patients with acute myocardial infarction had worse dental health than age- and sex-matched controls [8]. In addition, Oikarinen et al. revealed that radiographically diagnosed periodontal infection was more prevalent among patients with CAD than among control subjects [12]. Janket et al. also reported that the asymptotic dental score (a combined score for 5 oral pathologies) was significantly associated with CAD in their case-control study [6]. Furthermore, Paunio et al. reported that patients with ischaemic heart disease had more missing teeth than control subjects [9]. Sȍder et al. also showed that a high dental calculus score was associated with the incidence of angina pectoris in a cohort study comprised of 1676 patients [14]. A longitudinal study by DeStefano et al. demonstrated that subjects with periodontitis had a 25% higher risk of future coronary heart disease than subjects with minimal periodontal disease [11]. Similarly, a prospective 7-year follow-up study indicated that dental health significantly predicted coronary events among patients with coronary heart disease [7]. Finally, a meta-analysis of 9 studies revealed that periodontal disease was associated with a 19% increase in the risk of future cardiovascular disease among the general population [13]. Those results agree with our finding that tooth loss was independently associated with obstructive CAD. However, relative to previous studies that have focused on 1–2 dental health indices, our study examined 7 indices using a standardised protocol to minimise measurement error.

### Dental health examinations

Dental caries and periodontitis account for most infectious dental diseases [24], which makes the evaluation of these major oral diseases essential when quantifying dental health. The present study evaluated dental caries using the DFT and RNR, which are based on the mean numbers of decayed, missing, or filled teeth, as these are the most widely used methods for quantifying the degree of dental caries [22, 25]. The assessment of periodontitis is commonly based on the severity and extent of CAL and probing depth [26]. CPITN in this study was the modified community periodontal index of the WHO guideline and was measured through assessments of probing depth, gingival bleeding, and calculus. CPITN is one of the most credible measurement methods, enabling information comparisons at the national and global levels [22, 25, 27]. However, CPITN could overestimate the periodontal state, and may be limited in providing a detailed picture of severity [28]. Therefore, CAL was measured to compensate for these disadvantages. Furthermore, many previous studies have used dental caries and periodontitis to evaluate the relationship between dental infectious disease and systemic disease [6–8, 12, 13, 23]. Thus, the present study also included comprehensive dental indices, such as the TDI and PTI. Finally, we considered the total number of lost teeth, which is the most severe outcome of these dental infectious diseases. Therefore, we believe that we were able to evaluate the relationship of infectious dental disease with CAD at various stages of disease progression.

### Mechanisms

The underlying pathophysiology that could explain the relationship between poor dental health and CAD remains unclear, although dental infection may evoke a systemic inflammatory response that contributes to coronary atherosclerosis [4, 5]. In addition, periodontal disease increases the number of blood leukocytes and systemic levels of pro-inflammatory mediators, such as C-reactive protein, soluble cellular adhesion molecules, and fibrinogen, which are produced in response to cellular damage [29, 30]. In this context, tooth loss is the ultimate outcome of untreated dental disease and is a useful measure of oral health, because it reflects the cumulative effects of past disease and treatment [31, 32]. Furthermore, periodontal disease progresses slowly and is characterised by chronic infection and inflammation that leads to bone destruction and ultimately tooth loss [33]. Moreover, dental infection and CAD share some risk factors, such as smoking, diabetes, and low socioeconomic status [11, 34, 35].

### Study limitations

This study has several limitations. First, the sample size was small, which may explain why we failed to detect significant relationships between the other dental health indices and CAD. Second, the cross-sectional nature of this study precludes a conclusion regarding the causality of the relationship between tooth loss and obstructive CAD. Third, the present study did not consider socioeconomic status or dietary patterns, which can influence both dental health and coronary atherosclerosis.

## Conclusions

Tooth loss was associated with the presence of obstructive CAD in patients evaluated using coronary angiography. Larger well-designed longitudinal studies should be performed to determine whether there is a causal relationship between tooth loss and CAD.

## Abbreviations

ANOVA: Analysis of variance
BMI: Body mass index
CAD: Coronary artery disease
CAG: Coronary angiography
CAL: Clinical attachment loss
CCTA: Coronary computed tomography angiography
CPITN: Community periodontal index of treatment needs
DFT: Decayed and filled teeth
LDL: Low-density lipoprotein
PTI: Panoramic topography index
RNR: Ratio of no restoration
ROC: Receiver operating characteristic
TDI: Total dental index
